# Molecular Typing Characteristic and Drug Susceptibility Analysis of* Mycobacterium tuberculosis* Isolates from Zigong, China

**DOI:** 10.1155/2016/6790985

**Published:** 2016-02-11

**Authors:** Hai-Can Liu, Jian-Ping Deng, Hai-Yan Dong, Ti-Quan Xiao, Xiu-Qin Zhao, Zheng-Dong Zhang, Yi Jiang, Zhi-Guang Liu, Qun Li, Kang-Lin Wan

**Affiliations:** ^1^State Key Laboratory for Infectious Diseases Prevention and Control, Collaborative Innovation Center for Diagnosis and Treatment of Infectious Diseases, National Institute for Communicable Disease Control and Prevention, Chinese Center for Disease Control and Prevention, Beijing 102206, China; ^2^Zigong Center for Disease Control and Prevention, Zigong, Sichuan 643000, China

## Abstract

China is one of the 22 countries with high TB burden worldwide, and Sichuan contained the second-largest number of TB cases among all of the Chinese provinces. But the characteristics of* Mycobacterium tuberculosis* circulated in Zigong, Sichuan, were still unknown. To investigate the character and drug resistance profile, 265 clinical isolates were cultured from tuberculosis patient's sputum samples in the year of 2010, of which the genetic profile was determined by using Spoligotyping and MIRU-VNTR typing methods, and the drug sensibility testing to the four first-line and four second-line antituberculosis (anti-TB) drugs was performed by using proportion method on Lowenstein-Jensen (L-J) media. The major Spoligotype was Beijing family (143/265, 53.96%), followed by T (80/265, 30.19%) and H (9/265, 3.40%) genotypes; the total Hunter-Gaston discrimination index (HGDI) of the 24 loci MIRU-VNTR was 0.9995. About 27.17% (72/265) of the isolates were resistant to at least one of the eight tested anti-TB drugs, and for Beijing and non-Beijing family isolates the proportion of drug resistance was 28.47% (41/144) and 25.62% (31/121), respectively. That is, the most prevalent genotype here was Beijing family, and the 24 loci VNTR analysis could supply a high resolution for genotyping, and Beijing and non-Beijing isolates had no difference (*p* > 0.05) for drug resistance.

## 1. Introduction

Tuberculosis (TB) remains an important problem for the public health worldwide, especially in developing countries. Of the five countries with the largest number of incident cases in 2012, China (0.9 million–1.1 million) ranked the second just next to India, which accounted for 12% of the global cases alone [1]. Recently the emerging of drug-resistant TB (DR-TB) is becoming an important threat to TB control and public health worldwide [2, 3], and according to the National Survey of Drug-Resistant Tuberculosis in China, 5.7% of new TB patients and 25.6% of the previously treated cases were multi-drug-resistant TB (MDR-TB) [4]. In the last decade, genotyping of* Mycobacterium tuberculosis* (*M. tuberculosis*) has significantly enhanced our understanding of TB epidemiology by detecting suspected outbreaks and by tracing the infection routine. Such molecular methods like spacer oligonucleotide typing (Spoligotyping) and mycobacterial interspersed repetitive unit- (MIRU-) variable number tandem repeats (VNTR) typing have been used to characterize clinical* M. tuberculosis* isolates in China [5–7]. Obtaining the characteristic of* M. tuberculosis* isolates circulating in an area is helpful for understanding and controlling the spread of TB infection.

Although molecular epidemiology study of TB has been taken out in some area in China [8–10], few efforts have been made for the collection of DNA fingerprinting data based on the mutation profiles of isolates from the western region of China such as Sichuan province. Sichuan is a province that is located in southwestern China, with a population of approximately 87 million inhabitants. Sichuan contained the second-largest number of TB cases among Chinese provinces, where the prevalence of both TB and drug-resistant TB was much higher than the average level in China [11]. The total cases of TB in Sichuan province are estimated to be about 272,000 in 2006 and the new cases found in 2006 were more than 70,000 [12]. So the genetic and drug-resistant characteristics analysis of clinical* M. tuberculosis* isolates from this area may give us some benefits on TB control in China.

The aim of this study was to identify and genetically characterize the* M. tuberculosis* isolates circulating in Zigong, southern Sichuan, and to explore the distribution of drug resistance profiles across the major Spoligotyping-defined* M. tuberculosis* genotypes.

## 2. Material and Methods

### 2.1. Bacterial Strains and DNA Samples

A total of 265 clinical* M. tuberculosis* isolates were collected from Zigong, Sichuan, in 2010. Chromosomal DNA was extracted from fresh bacteria cultures on the Lowenstein-Jensen (L-J) medium. A loopful of mycobacterial colonies were suspended in 400 *μ*L TE buffer (pH 8.0) and then boiled for 10 min. The suspension was centrifuged at 12,000 rpm for 10 min, and the supernatant was served as PCR template and stored at −20°C.

### 2.2. Spoligotyping

Spoligotyping was performed as described previously by Kamerbeek et al. [13]. The results were entered into Excel spreadsheets in a binary format and compared with SITVITWEB database [14], which is a publicly available international multimarker database for studying* Mycobacterium*.

### 2.3. MIRU-VNTR Typing

VNTR analysis was performed by the amplification of 24 genomic loci, the scheme of which has been described by Supply et al. [15]. Each VNTR locus was amplified individually in 20 *μ*L reaction mixture containing 1x PCR GC Buffer I (Takara Bio Inc.), 5 pmol of each primer set, 200 *μ*M of each dNTP, 0.5 U of DNA Taq Polymerase (Takara Bio Inc.), and 1.5 ng of DNA template. An initial denaturation step of 5 min at 95°C was followed by 35 cycles at 94°C for 1 min, 60°C for 1 min, and 72°C for 1 min, with a final extension at 72°C for 10 min. The PCR products were analyzed by electrophoresis on 2% agarose gel using 100 bp DNA ladder as size markers. The H37Rv strain was included as a positive control for each amplification reaction and run as an additional control for accuracy, and then the copy number of each locus was calculated by using the Image Lab software (Bio-Rad).

### 2.4. Drug Susceptibility Testing

Drug susceptibility testing of those 265 strains to the four first-line drugs (rifampicin [RFP], isoniazid [INH], streptomycin [SM], and ethambutol [EMB]) and four second-line drugs (amikacin [AMK], kanamycin [KA], ofloxacin [OFLX], and capreomycin [CPM]) was performed by using proportion method recommended by WHO [16]. Multi-drug-resistant TB (MDR-TB) is the isolate that was resistant to at least the two first-line anti-TB drugs, RFP and INH, based on the WHO definition [17].

### 2.5. Computer Analysis

The Spoligotyping and MIRU-VNTR typing results of all the* M. tuberculosis* clinical isolates were analyzed by the BioNumerics program (Windows 7, version 5.10; Applied Maths, Kortrijk, Belgium). Clusters were defined as groups that the* M. tuberculosis* isolates have identical Spoligotyping or MIRU-VNTR patterns. The dendrograms based on the Spoligotyping and 24 VNTR loci data were conducted using UPGMA protocol. The Hunter-Gaston discrimination index (HGDI) for the 24 VNTR loci was calculated using the previously reported formula [18]. The HGDI of each VNTR locus was calculated by using the web based VNTR Diversity and Confidence Extractor (V-DICE http://www.hpa-bioinformatics.org.uk/cgi-bin/DICI/DICI.pl).

### 2.6. Statistical Analysis

Chi-squared tests were used to compare the proportions of DR-TB and the distribution of genotypes in different groups. All tests of significance were two-sided and the significant threshold was set at 0.05.

## 3. Results

### 3.1. Demographic Characters

All of the 265 clinical isolates were cultured from sputum samples of different TB patients in Zigong CDC, Sichuan province, in 2010. And for the 265 TB patients, 44 (16.60%) were previously treated, 139 (52.45%) were in the 30- and 60-year-old age group, 39 (14.72%) female and 209 (78.87%) male patients with other 17 (6.41%) gender uncertain patients, and no difference about these factors between Beijing and non-Beijing family strains was found at *p* < 0.05 level (Table 1).

### 3.2. Spoligotyping

**Figure 1:**
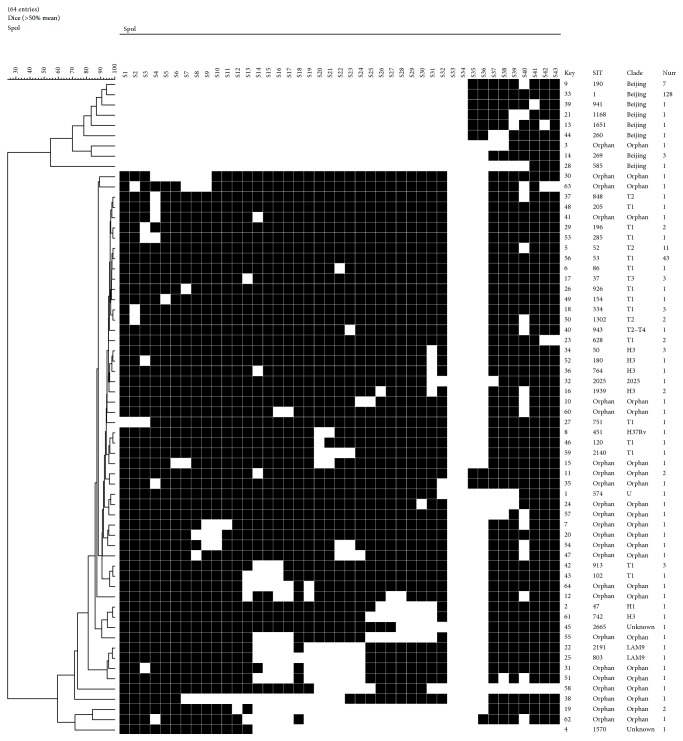


Based on the Spoligotyping results, all of the 265 isolates were subdivided into 64 Spoligotypes, among which 49 patterns were unique whereas other 216 isolates were grouped into 15 clusters containing 2 to 128 isolates. Following comparison with the SITVITWEB database, 239 isolates were clustered into 40 shared international types (SITs), whereas the other 26 isolates that had not been described in the database were referred to as “Orphan.” Family assignment revealed that 143 strains had the classical Beijing family-specific or Beijing family-like Spoligotyping pattern. We treated all these 143 isolates as Beijing family strains (Figure 1). In total, the Beijing family is clearly the most prevalent genetic family in our research setting (143/265, 53.96%), followed by T family (80/265, 30.19%) and H family (9/265, 3.40%).

### 3.3. MIRU-VNTR Typing

**Figure 2:**
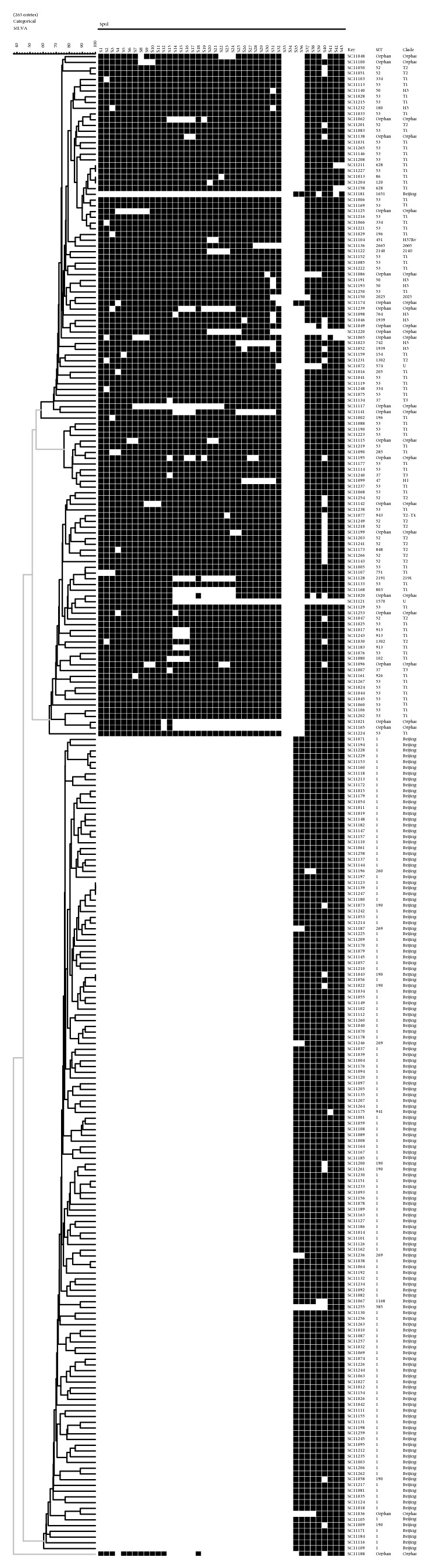


Exploitable VNTR results (defined as less than 4 missing values) were obtained for 262 isolates out of 265 isolates. Using the 24 polymorphic loci, all of the 262* M. tuberculosis* isolates were identified to 257 different VNTR types and 3 main clusters. A total of 253 isolates had unique profiles. The remaining 9 isolates formed 4 clusters (2 to 3 isolates) (Figure 2). The total Hunter-Gaston discrimination index (HGDI) for all of the 263 isolates was 0.9995, and that of Beijing and non-Beijing family isolates was 0.9987 and 0.9992, respectively; the HGDI of each of the 24 VNTR loci were shown in Table 2.

### 3.4. Drug-Resistant Phenotypic Profiles

Among all the 265 isolates, 72 (27.17%) were resistant to at least one of the eight tested anti-TB drugs, and other 193 (72.83%) were sensitive. Of the 72 drug-resistant isolates, 48 (18.11%) were INH-resistant, 40 (15.09%) were RIF-resistant, 32 (12.08%) were SM-resistant, 17 (6.42%) were EMB-resistant, 32 (12.08%) were OFLX-resistant, and 36 (13.58%) of those drug-resistant strains were MDR-TB; no XDR-TB was found in this study (Table 3). When grouped by gender or age group like previous report [8], no relationship was found with the isolate's character of drug resistance. But having been treated before may play an important role with the drug resistance, not only for the drug-resistant TB (*χ* ^2^ = 11.2680, *p* < 0.05) but also for the MDR-TB (*χ* ^2^ = 18.8983, *p* < 0.05) (Table 4).

### 3.5. Relationship between Beijing Family and Drug Resistance

According to the demographic data, no matter whether they are grouped by gender, age groups, or even treatment history, there was no difference of the infection rate between Beijing and non-Beijing family isolates observed (Table 1). And for the Spoligotyping patterns and DST results, of the 143 Beijing family isolates, 41 (28.67%) were drug-resistant to at least one of the eight anti-TB drugs of which 23 (16.08%) were MDR-TB. While, in the 122 non-Beijing isolates, 31 (25.41%) were drug-resistant and 13 (10.66%) were MDR-TB, no difference of the drug resistance proportion was found between Beijing and non-Beijing family isolates (Table 3).

## 4. Discussion

Previous research report has reviewed that Beijing family is prevalent (57.89%) in Sichuan province [6], and according to the Spoligotyping results in this study, the proportion of Beijing family is about 54.34% in southern Sichuan, a little lower than that of the previous report. This may be because our study was just focused on the prevalence isolates in Zigong, and more isolates were included in this study which could afford much more representativeness. But this still conforms to the former reports that the most prevalent isolate here was Beijing family strains and the highest prevalence of Beijing family was found in northern China, followed by central and southern China [6, 7]. Besides, T genotype was the second major epidemic genotype circulating in this area and was also consistent with the previous study [19]. This may indicate that non-Beijing family strains also played an important role in the high tuberculosis burden in Sichuan and some other provinces that are located in southern China [6, 7].

Spoligotyping has been considered as the gold standard for Beijing family identification, as it is simple and efficient. But the discrimination of Spoligotypes for Beijing family is very low and it cannot further differentiate Beijing family strains, as Beijing family strains always show characteristic homological Spoligotyping pattern [20]. For the 24 loci MIRU-VNTR typing method, even the discrimination of each locus is different, but when we combined them together a high resolution was acquired (Table 2). As the MIRU-VNTR genotyping is considered to be faster and easier to perform, the results are in digital format which can be easily compared between laboratories. Thus, if conditions allow, we suggest Spoligotyping and MIRU-VNTR genotyping should be taken out at the same time, to investigate in more detail characters of the selected isolates. But for some loci the HGDI were so low; different countries could select specific loci for genotyping. And for the three main clusters of the UPGMA dendrogram in Figure 2, one formed by Beijing family isolates and other two within non-Beijing family isolates, but no difference was found between them for difference factors, like drug resistance and patient age group.

In our study, 27.17% (*n* = 72) of the isolates were resistant to at least one of the eight anti-TB drugs, and 13.58% (*n* = 36) were MDR-TB; no XDR-TB was found in this study. These have some difference with the whole nation's status [4]. Firstly, we focused on the target area here in Sichuan province; the demographic and* M. tuberculosis* infectious status in this area may have some differences with those of the whole nation. Secondly, the isolates selected into our study may also have some differences with that of the previously report. Also in our study we found that the treatment history may play an important role with the drug resistance, not only for drug-resistant TB but also for MDR-TB (Table 4); thus the management of TB patients should be strengthened. And as the drug-resistant TB may be the most important challenge to achieve the goals of stopping TB, much more attention should be paid to it.

As Beijing family strains were prevalent in this area (143/265, 53.96%) and in some reports, Beijing family has been considered to be related to TB outbreak and drug resistance [21–24], while some are not [25–27]. Referring to our study, the chi-square analysis showed that there was no significant difference between the Beijing and non-Beijing family strains in terms of their drug resistance proportion and patient age group. This may be because Beijing family is the genetic aspect but the drug resistance is a biological aspect of one* M. tuberculosis* isolate, and there is no constant relationship between them.

## Figures and Tables

**Table 1:** Demographic data of the 265 isolates in Zigong, China.

Factors	Whole (*n* = 265)	Beijing (*n* = 143)	Non-Beijing (*n* = 122)	*χ* ^2^^*∗*^ (*p* value)
Sex				
Female	39	25	14	1.6036 (0.2054)
Male	209	111	98	
Unknown	17	7	10	
Age				
<30	27	19	8	6.9592 (0.0732)
*⩾*30	139	79	60	
*⩾*60	69	30	39	
*⩾*75	9	6	3	
Unknown	21	9	12	
Treated before?				
Yes	44	25	19	0.1732 (0.6773)
No	221	118	103	

*∗*: evaluated the difference between Beijing and non-Beijing family strains.

**Table 2:** HGDI of the 24 MIRU-VNTR loci for the whole sample, Beijing and non-Beijing family isolates.

Locus	Whole (*n* = 265)	Beijing (*n* = 143)	Non-Beijing (*n* = 122)
ETRA	0.594	0.262	0.606
ETRB	0.374	0.068	0.504
ETRC	0.110	0.069	0.159
ETRD	0.303	0.028	0.540
ETRE	0.592	0.245	0.202
MIRU02	0.030	0.000	0.065
MIRU10	0.576	0.263	0.242
MIRU16	0.283	0.181	0.392
MIRU20	0.246	0.240	0.254
MIRU23	0.124	0.108	0.143
MIRU24	0.015	0.014	0.017
MIRU26	0.739	0.570	0.598
MIRU27	0.328	0.028	0.523
MIRU39	0.521	0.082	0.097
MIRU40	0.551	0.485	0.619
Mtub04	0.620	0.363	0.622
Mtub21	0.806	0.710	0.571
Mtub29	0.096	0.069	0.127
Mtub30	0.496	0.119	0.182
Mtub34	0.095	0.042	0.157
Mtub39	0.511	0.267	0.647
QUB11b	0.854	0.745	0.796
QUB26	0.723	0.571	0.821
QUB4156	0.052	0.081	0.017

**Table 3:** Prevalence of anti-TB drug resistance among 265 isolates in Sichuan province^*∗*^.

Anti-TB drugs	Whole (*n* = 265)	Beijing (*n* = 143)	Non-Beijing (*n* = 122)	*χ* ^2^ (*p* value)
Isoniazid	18.11% (*n* = 48)	19.44% (*n* = 28)	16.53% (*n* = 20)	0.45 (0.5019)
Rifampicin	15.09% (*n* = 40)	18.75% (*n* = 27)	10.74% (*n* = 13)	3.48 (0.0623)
Streptomycin	12.08% (*n* = 32)	13.89% (*n* = 20)	9.92% (*n* = 12)	1.07 (0.3014)
Ethambutol	6.42% (*n* = 17)	7.64% (*n* = 11)	4.96% (*n* = 6)	0.84 (0.3583)
Ofloxacin	12.08% (*n* = 32)	9.03% (*n* = 13)	15.70% (*n* = 19)	2.61 (0.1065)
Resistant to any anti-TB drugs	27.17% (*n* = 72)	28.47% (*n* = 41)	25.62 % (*n* = 31)	0.35 (0.5519)
MDR-TB	13.58% (*n* = 36)	15.97% (*n* = 23)	10.74% (*n* = 13)	1.65 (0.1986)

*∗*: all 265 isolates were sensitive to other three kinds of anti-TB drugs, and Pearson *χ* ^2^ and *p* value were evaluated between the Beijing and non-Beijing family strains.

**Table 4:** Prevalence of anti-TB drug resistance among 265 isolates by factors.

Factors	Sensitive TB (*n* = 193)	Any drug resistance TB (*n* = 72)	*χ* ^2^ (*p* value)	Non-MDR-TB (*n* = 229)	MDR-TB (*n* = 36)	*χ* ^2^ (*p* value)
Sex						
Female	29	10	0.1097 (0.7405)	34	5	0.0095 (0.9225)
Male	150	59		181	28	
Unknown	14	3		14	3	
Age						
<30	22	5	4.1444^*∗*^ (0.2402)	23	4	4.8455^*∗*^ (0.1579)
*⩾*30	93	46		115	24	
*⩾*60	52	17		64	5	
*⩾*75	8	1		9	0	
Unknown	18	3		18	3	
Treated?						
Yes	23	21	11.2680 (0.0008)	29	15	18.8983 (0.0000)
No	170	51		200	21	

*∗*: evaluated by the Fisher's exact test.

## References

[1] World Health Organization (2013). *Global Tuberculosis Report 2013*.

[2] Gandhi N. R., Nunn P., Dheda K. (2010). Multidrug-resistant and extensively drug-resistant tuberculosis: a threat to global control of tuberculosis. *The Lancet*.

[3] World Health Organization (2010). *Multidrug and Extensively Drug-Resistant TB (M/XDR-TB): 2010 Global Report on Surveillance and Response*.

[4] Zhao Y., Xu S., Wang L. (2012). National survey of drug-resistant tuberculosis in China. *The New England Journal of Medicine*.

[5] Jiang Y., Liu H.-C., Zheng H. (2013). 19-VNTR loci used in genotyping Chinese clinical *Mycobacterium tuberculosis* complex strains and in association with spoligotyping. *Journal of Basic Microbiology*.

[6] Dong H., Liu Z., Lv B. (2010). Spoligotypes of Mycobacterium tuberculosis from different provinces of China. *Journal of Clinical Microbiology*.

[7] Dong H., Shi L., Zhao X. (2012). Genetic diversity of *Mycobacterium tuberculosis* isolates from Tibetans in Tibet, China. *PLoS ONE*.

[8] Liu Q., Yang D., Xu W. (2011). Molecular typing of *Mycobacterium tuberculosis* isolates circulating in Jiangsu province, China. *BMC Infectious Diseases*.

[9] Lu B., Zhao P., Liu B. (2012). Genetic diversity of Mycobacterium tuberculosis isolates from Beijing, China assessed by Spoligotyping, LSPs and VNTR profiles. *BMC Infectious Diseases*.

[10] Yu Q., Su Y., Lu B. (2013). Genetic diversity of *Mycobacterium tuberculosis* isolates from Inner Mongolia, China. *PLoS ONE*.

[11] Wang L., Cheng S., Chen M. (2012). The fifth national tuberculosis epidemiological survey in 2010. *Chinese Journal of Antituberculosis*.

[12] Guo J.-H., Xiang W.-L., Zhao Q.-R. (2008). Molecular characterization of drug-resistant *Mycobacterium tuberculosis* isolates from Sichuan Province in China. *Japanese Journal of Infectious Diseases*.

[13] Kamerbeek J., Schouls L., Kolk A. (1997). Simultaneous detection and strain differentiation of *Mycobacterium tuberculosis* for diagnosis and epidemiology. *Journal of Clinical Microbiology*.

[14] Demay C., Liens B., Burguière T. (2012). SITVITWEB—a publicly available international multimarker database for studying *Mycobacterium tuberculosis* genetic diversity and molecular epidemiology. *Infection, Genetics and Evolution*.

[15] Supply P., Mazars E., Lesjean S., Vincent V., Gicquel B., Locht C. (2000). Variable human minisatellite-like regions in the *Mycobacterium tuberculosis* genome. *Molecular Microbiology*.

[16] World Health Organization (2009). *Guidelines for Surveillance of Drug Resistance in Tuberculosis*.

[17] World Health Organization (2011). *Guidelines for the Programmatic Management of Drug-Resistant Tuberculosis-2011 Update*.

[18] Hunter P. R., Gaston M. A. (1988). Numerical index of the discriminatory ability of typing systems: an application of Simpson's index of diversity. *Journal of Clinical Microbiology*.

[19] Wan K., Liu J., Hauck Y. (2011). Investigation on *Mycobacterium tuberculosis* diversity in china and the origin of the Beijing clade. *PLoS ONE*.

[20] van Soolingen D., Qian L., de Haas P. E. W. (1995). Predominance of a single genotype of *Mycobacterium tuberculosis* in countries of east Asia. *Journal of Clinical Microbiology*.

[21] Kremer K., Glynn J. R., Lillebaek T. (2004). Definition of the Beijing/W lineage of *Mycobacterium tuberculosis* on the basis of genetic markers. *Journal of Clinical Microbiology*.

[22] Hu Y., Ma X., Graviss E. A., Wang W., Jiang W., Xu B. (2011). A major subgroup of Beijing family *Mycobacterium tuberculosis* is associated with multidrug resistance and increased transmissibility. *Epidemiology and Infection*.

[23] Purwar S., Chaudhari S., Katoch V. M. (2011). Determination of drug susceptibility patterns and genotypes of *Mycobacterium tuberculosis* isolates from Kanpur district, North India. *Infection, Genetics and Evolution*.

[24] Brown T., Nikolayevskyy V., Velji P., Drobniewski F. (2010). Associations between *Mycobacterium tuberculosis* strains and phenotypes. *Emerging Infectious Diseases*.

[25] Ani A., Bruvik T., Okoh Y. (2010). Genetic diversity of *Mycobacterium tuberculosis* complex in Jos, Nigeria. *BMC Infectious Diseases*.

[26] Kai M. K., Chi W. Y., Lai W. T. (2005). Utility of mycobacterial interspersed repetitive unit typing for differentiating multidrug-resistant *Mycobacterium tuberculosis* isolates of the Beijing family. *Journal of Clinical Microbiology*.

[27] Zhang J., Mi L., Wang Y. (2012). Genotypes and drug susceptibility of *Mycobacterium tuberculosis* Isolates in Shihezi, Xinjiang Province, China. *BMC Research Notes*.

